# Supplementary material for: MetaRibo-Seq measures translation in microbiomes
Source: Nat Commun. 2020 Jun 29;11:3268. doi: 10.1038/s41467-020-17081-z (PMC7324362; doi:10.1038/s41467-020-17081-z)
Supplement: Supplementary file 10 — Supplementary Data 7 [file 41467_2020_17081_MOESM10_ESM.zip › File2/Confidence_High_Taxonomy/139857_out.krona.html]

Javascript must be enabled to view this page.

members
magnitude
magnitudeUnassigned
count
unassigned
taxon
rank

139857\_out

2

1
superkingdom
2

phylum
1
1239

class
1
186801

186802
1
order

186803
1
family

species
1
1952077

SRS019798\_contig\_number\_contig-100\_13180.13181

1

SRS015431\_contig\_number\_contig-100\_39591.126745
